# Heart Failure Status among Acute Ischemic Stroke Patients: A Hospital-Based Study

**DOI:** 10.1155/2022/7348505

**Published:** 2022-08-24

**Authors:** Fatemeh Ravandi, Arsh Haj Mohamad Ebrahim Ketabforoush, Fereshteh Azedi, Mohsen Hoshyarkhani, Farimah Fayyaz, Nahid Abbasi Khoshsirat

**Affiliations:** ^1^Student Research Committee, Alborz University of Medical Sciences, Karaj, Iran; ^2^Cellular & Molecular Research Center, Iran University of Medical Sciences, Tehran, Iran; ^3^Department of Neurology, Shahid Rajaei Clinical Research and Development Unit, Alborz University of Medical Sciences, Karaj, Iran

## Abstract

**Background:**

Since heart failure (HF) and ischemic stroke have common risk factors, their concurrent occurrence is likely. Strokes in HF patients could be life-threatening and lead to severe disabilities, longer hospitalization time, and mortality. The present study aims to investigate the prevalence of HF and its severity based on ejection fraction (EF) in patients with acute ischemic stroke.

**Methods:**

The present cross-sectional study included acute ischemic stroke patients admitted to Shahid Rajaei hospital in Karaj in 2020–2021. The diagnosis of HF was based on transthoracic echocardiography within 48 hours of symptom onset, and HF was classified into two groups: 41–49% as mildly reduced EF (HFmrEF) and ≤40% as reduced EF (HFrEF). Patients who did not complete cardiac studies were excluded.

**Results:**

257 acute ischemic stroke patients (62.6% male) were included. Among stroke patients, the prevalence of HF, including HFrEF and HFmrEF, was 30.0% (95% CI: 21.4–38.6). HFmrEF and HFrEF was diagnosed in 32 (12.5%) and 45 (17.5%) patients, respectively. HF was significantly associated with older age, hypertension, past myocardial infarction (MI), and arrhythmia. A history of previous MI significantly increased the odds of heart failure (OR: 3.25, 95% CI: 1.82–5.81).

**Conclusion:**

There is a high prevalence of HF among acute ischemic stroke patients. Older patients with a history of hypertension and previous MI are at higher risk. Since patients with HF have a higher mortality and morbidity rate after experiencing an ischemic stroke, close cooperation between the neurology and cardiology specialists for providing advanced care for survivors is required.

## 1. Introduction

Heart Failure (HF) is one of the leading causes of impairments and global health issues [1]. It is a clinical syndrome consisting of major symptoms and signs, including shortness of breath, fatigue, swelling of lower limbs, elevated jugular venous pressure, and pulmonary and peripheral edema [2]. According to the left ventricular ejection fraction (LVEF), HF is classified into three groups: reduced LVEF (HFrEF), defined as LVEF ≤ 40%, mildly reduced LVEF (HFmrEF) with LVEF between 41% and 49%, and preserved LVEF (HFpEF) in which patients have LVEF ≥ 50% [3]. It is estimated that the global prevalence of HF is 64.3 million people [1]. The lifetime risk of HF from the age of 45 years through the age 95 of years is reported to be 30–42% in white men, 20–29% in black men, 32–39% in white women, and 24–46% in black women [4, 5].

Cerebrovascular accident (CVA) is divided into ischemic and hemorrhagic types. While hemorrhagic stroke is due to intracranial or subarachnoid hemorrhage, ischemic stroke is caused by thrombosis or embolism. The global prevalence of CVA is 101.5 million people, among which ischemic stroke compromised 77.2 million people. Moreover, the ischemic stroke age-standardized prevalence rate had a 3.6% increase from 2010 to 2019. In addition, despite the 14.7% decrease in the age-standardized mortality rate between 2010 and 2019, the absolute number of stroke deaths had a 12.2% increase in the same period [6]. In 2010, 39.4 million and 62.8 million disability-adjusted life-years (DALYs) were lost due to ischemic and hemorrhagic stroke, respectively [7, 8].

Atrial fibrillation (AF) is one of the main risk factors for cardioembolic CVAs. The prevalence of strokes associated with AF increased threefold in the past three decades [9]. Due to the common risk factors of HF and CVA, the concurrent occurrence of these two conditions is not rare. It has been reported that the risk of ischemic stroke in HF patients is two–three times higher than that in people without HF [10]. Ischemic strokes in HF patients could be life-threatening, leading to severe disabilities, longer hospitalization time, and mortality [11–14]. Since AF is one of the main risk factors for stroke, anticoagulants, including warfarin and direct-acting oral anticoagulants (DOACs), can be recommended in this group of patients [15]. Although anticoagulants need monitoring and are costly, their benefit could not be disregarded due to their ability to prevent stroke recurrence and the following physical disabilities. Therefore, in the present study, we investigate the prevalence of HF and its severity based on EF in patients with ischemic stroke.

## 2. Methods

In the present cross-sectional study, all cases with ischemic stroke admitted to the Neurology Department of Shahid Rajaei Hospital in Karaj from December 2020 to December 2021 were included. A stroke was described as persisting of an acute neurologic deficit for more than 24 hours due to a vascular cause with no signs of hemorrhage or other reasons. At least two neurologists have established the diagnosis of ischemic stroke based on the Trial of Org 10172 in Acute Stroke Treatment (TOAST) classification [16] with the accompaniment of brain imaging, including a brain CT scan or MRI. All participants were assessed by a uniform protocol based on the American Stroke Association guidelines [17]. We obtained the demographic factors and past medical history containing important vascular risk factors from medical records, patients, and their relatives. The definitions that we used were as follows: hypertension (measuring at least two arterial blood pressure documented with a range of diastolic >90 or systolic >140 mmHg on different days or one week after stroke inception or previous medication consumption); diabetes (FBS ≥ 126 mg/dl or the use of relevant medications); hyperlipidemia (serum cholesterol level >200 mg/dl or serum triglyceride level >150 mg/dl or medication usage); ischemic heart disease (verified history of myocardial infarction and/or angina pectoris); arrhythmia (chronic or paroxysmal atrial fibrillation established by ECG); anticoagulant usage before stroke onset; and a previous history of stroke. Transthoracic echocardiography (TTE) was performed for all cases within 48 hours of symptom onset as a part of a standard stroke evaluation protocol. The diagnosis of HF was achieved by a trained cardiologist using a 2.5-MHz transducer using M-mode, 2-dimensional, and Doppler evaluation, according to the recommendations of the American Echocardiography Society [18]. The severity of HF using left ventricle ejection fraction (LVEF) reports was classified into two groups: 41–49% as mildly reduced EF (HFmrEF) and ≤40% as reduced EF (HFrEF). Patients who were under 18 years of age and did not have complete and adequate cardiac study, including EF measurement from TTE, were excluded.

Data were analyzed using SPSS version 26.0 (SPSS Inc., IBM Company). Initially, the normal distribution of data was determined by the Lilliefors test based on the Kolmogorov–Smirnov test. The quantitative variables were reported as mean ± standard deviation (SD) or median and interquartile range (IQR), while the qualitative variables were reported as frequency and percentage. The independent-sample *t* and one-way ANOVA tests were used to analyze parametrically and the Mann–Whitney U and Kruskal–Wallis tests for nonparametric quantitative variables. Pearson's chi-square tests assessed the association between qualitative variables. The logistic regression test was used to determine the degree of association of demographic and risk factors with heart failure. All variables with a *P*-value < 0.2 in the univariate analysis were presented to the multiple logistic regression model (crude model). An adjusted model was used to adjust for the presence of confounding factors. The results of logistic regression analysis are presented by OR and 95% confidence interval. A *P*-value less than 0.05 was considered statistically significant.

This study was reviewed and approved by the Ethical Committee of the Alborz University of Medical Sciences (Code No. IR.ABZUMS.REC.1399.331). Also, informed consent was obtained from the patients or their relatives.

## 3. Results

In the present study, 281 patients presented to Rajaei hospital in Karaj with acute ischemic stroke manifestations, of which 24 were excluded from analysis due to the lack of TTE and incomplete cardiac study. The remaining 257 ischemic stroke patients were included. 161 patients (62.6%) were male and 96 patients (37.4%) were female.

The median (interquartile range) age of patients was 65 [18] years. Regarding the past medical and drug history of patients, 44 (17.1%) hyperlipidemic patients, 178 (69.3%) hypertensive patients, 101 (39.3%) diabetic patients, 95 (37.0%) patients with a past myocardial infarction (MI) history, 56 (21.8%) patients with the previous stroke, and 31 (12.1%) patients with arrhythmia were reported, and 11 (4.3%) patients used anticoagulants.

It was demonstrated that the mean (SD) EF was 48.49% (±9.94), ranging from 10% to 60%. Among the stroke patients, the prevalence of HF, including HFrEF and HFmrEF, was 30.0% ((21.4–38.6); CI 95%). HFmrEF and HFrEF were diagnosed in 32 (12.5%) and 45 (17.5%) patients, respectively.

The association of the HF rate and its severity classifications with age, gender, hyperlipidemia, hypertension, diabetes, past MI and stroke history, arrhythmia, anticoagulant consumption, and duration of hospitalization was investigated (Table 1). Age was correlated with the HF rate (*P*=0.012) and severity classifications (*P*=0.02). Older ischemic stroke patients were more likely to have HF. Moreover, gender did not correlate with the HF rate or its severity. Among the studied past medical history variables, the history of hypertension, past MI, and arrhythmia were associated with higher HF rates (*P*=0.01, *P* < 0.01, and *P*=0.02, respectively). Also, the severity of HF was correlated with a history of hypertension, past MI, and arrhythmia (*P*=0.03, *P* < 0.01, and *P*=0.02, respectively). The duration of hospitalization was not associated with the HF rate in ischemic stroke patients (*P*=0.15).

Table 2 demonstrated the association of demographic and risk factor variables with heart failure in logistic regression analysis among the studied ischemic stroke patients. In the crude model, by increasing age per year, the odds of heart failure increased by 3% (OR: 1.03, 95% CI: 1.01–1.05; *P*-value = 0.01). Also, hypertension, past MI history, and arrhythmia were significantly higher in the patients with HF (OR: 2.28, 95% CI: 1.20–4.33; OR: 3.97, 95% CI: 3.97, 95% CI: 2.26–6.96; OR: 2.48, 95% CI: 1.16–5.32, respectively). In the adjusted model, a history of previous MI significantly increased the odds of heart failure (OR:3.25, 95% CI: 1.82–5.81; *P*-value <0.001).

## 4. Discussion

The findings of the present study demonstrated that 30% of patients with ischemic CVA suffered from HF. Also, 12.5% of patients had HFmrEF and 17.5% had HFrEF. The mean (SD) EF was 48.49% (±9.94), ranging from 10% to 60%. Several studies have investigated the prevalence of HF among stroke patients. Appelros et al. evaluated 377 patients with first-ever stroke (excluding subarachnoid hemorrhage patients) in a cohort. They demonstrated that 14% of patients had HF, which was associated with dependency along with age and stroke severity [19]. In a case-control study, 270 patients with first ischemic stroke and 288 demographically-matched controls were evaluated. The rate of HF was significantly higher in the case group (24.1% vs. 4.9%; *P* < 0.0001). In addition, the adjusted odds ratio for HF was 3.92 (1.93–7.92; 95% CI) [20]. Ois et al. showed that 17.7% of 503 patients with ischemic stroke had HF, 9.7% with systolic HF, and 8% with preserved LVEF. HF was significantly associated with older age, higher initial stroke severity, lower rate of current smoking habits, and higher rates of antithrombotic pretreatment, ischemic heart disease, peripheral arterial disease, AF, and lacunar stroke. Additionally, the outcome of patients after 90 days from stroke onset showed that systolic HF and HFpEF were associated with poor outcomes [21].

In the present study, HF was associated with older age, hypertension, past MI history, and arrhythmia. The retrospective analysis of stroke patients in 1995 and 2005 in the United States showed that the rate of HF was 10.8% in 1995 and 12.3% in 2005. Stroke patients with HF were more likely to be female, smokers, or older and had higher rates of ischemic heart disease, diabetes, hypertension, and AF than stroke patients without HF. Moreover, stroke patients with HF had a higher in-hospital mortality rate and hospitalization period and needed more intensive care, including mechanical ventilation, gastrostomy, and tracheostomy, than those without HF [12]. In a study by Palumbo et al. the records of 130 patients with acute ischemic stroke were retrospectively reviewed. Seventeen patients (13.1%) had a history of HF, and the mean EF (SD) was 54.8% (±10.4). In multivariable analysis, the LVEF<40% and clinical diagnosis of HF could predict the 90-day mortality rate [22]. In another study, among 566 patients with ischemic stroke, 96 patients (17%) were diagnosed with HF, 21 patients with HFrEF, and 55 with HFpEF. The factors significantly associated with HF in stroke patients included older age, hypertension, diabetes, previous MI, and AF. The adjusted odds ratio of HF as a predictor of poor outcome was 2.34 (1.12–4.89; *P*=0.02, 95% CI) [23]. In another retrospective study, Li et al. analyzed 685 patients with acute ischemic stroke and showed that 86 patients (12.6%) had left ventricular systolic dysfunction [13]. In a large study in Thailand between 2004 and 2015, among 370,527 patients with acute ischemic stroke, 11,522 patients (3.1%) had HF. The follow-up of patients for a median of 4.47 years demonstrated that HF was associated with higher postdischarge mortality [14]. In a prospective cohort of 10,816 acute ischemic stroke patients at a center in the UK, 1,543 patients (14.3%) suffered from HF. HF was associated with increased in-hospital mortality and length of stay. The odds ratio of long-term mortality after 5.5 years follow up and recurrent stroke after 3.7 years follow up was increased and significant [11]. Among 1,209 patients with acute ischemic stroke, 378 patients (31%) had HF. 25 patients (7%) with preserved LVEF, 228 (60%) with 35% < EF < 50%, 33 (9%) with 25% ≤EF ≤ 35%, and 44 (12%) with EF<25% [24]. In a prospective cohort study between 2014 and 2017, 644 ischemic stroke patients were included, among which HF was present in 35 patients (5.4%). The prevalence of HF classifications was HFpEF 4.35%, HFmrEF 0.31%, and HFrEF 0.78%. Patients with HF were more likely to be female, older, and have AF and hypertension [25].

Although the presence of AF is a known risk factor for stroke in patients with HF, likely, HF could also increase the risk of stroke in the absence of AF [26–28]. The suggested mechanisms of stroke in HF without AF include thromboembolism due to blood stasis and prothrombotic state, hypoperfusion, and concomitant arterial diseases [29]. However, in our study, previous anticoagulant consumption does not differ significantly between patients with and without HF; anticoagulation therapy with the aim of stroke prevention in HF patients is controversial. Treatment with OACs in HF patients with sinus rhythm has been investigated in several studies. By considering the increased risk of hemorrhage with anticoagulation therapies, the use of OACs should be individualized based on the additional indications and the benefit of stroke prevention. Moreover, the use of DOACs that have lower adverse effects and are easier to administer needs to be further investigated in stroke patients with HF [30–35].

In conclusion, we had the following limitations: besides our reasonable population size due to the single-center design, our findings cannot be generalized to other healthcare centers or medical settings. We have not evaluated diastolic function; therefore, there may be preserved EF HF patients with or without diastolic dysfunction who may not have common HF symptoms. Additionally, in the setting of acute ischemic stroke, reduced EF HF patients may be a temporary condition as a result of autonomic disruption and stress-induced cardiomyopathy.

More excellent knowledge about HF can aid medical staff in making more reasonable clinical decisions to enhance the outcome of stroke patients with this state. Based on that, we recommend future studies to analyze other echocardiographic HF markers, such as the effect of diastolic function in a prospective method and assess the outcome of HF patients based on the severity of the stroke.

## 5. Conclusion

Our findings and previous studies show a high prevalence of HF among acute ischemic stroke patients. This association depends on some of the common risk factors for these two diseases. Older patients with a history of hypertension and previous MI are at higher risk. Since patients with HF have higher mortality and morbidity rate after experiencing an ischemic stroke, close cooperation between neurology and cardiology specialists for providing advanced care for survivors is required.

## Figures and Tables

**Table 1:** Heart failure and its severity association with demographic, and past medical and drug histories of ischemic stroke patients.

		No Heart Failure (EF ≥ 50%)	Heart Failure (EF < 50%)	*P*-value	Heart Failure severity		*P*-value
					HFmrEF	HFrEF	
Age, years; mean (SD)		65.26 (13.18)	69.79 (12.25)	0.01^*∗*^	72.44 (14.58)	67.91 (10.02)	0.02^*∗∗*^

Gender; N (%)	Male	110 (68.3%)	51 (31.7%)	0.44	19 (11.8%)	32 (19.9%)	0.43
	Female	70 (72.9%)	26 (27.1%)		13 (13.5%)	13 (13.5%)	

Hyperlipidemia; N (%)	Yes	26 (59.1%)	18 (40.9%)	0.08	8 (18.2%)	10 (22.7%)	0.21
	No	154 (72.3%)	59 (27.7%)		24 (11.3%)	35 (16.4%)	

Hypertension; N (%)	Yes	116 (65.2%)	62 (34.8%)	0.01	27 (15.2%)	35 (19.7%)	0.03
	No	64 (81.0%)	15 (19.0%)		5 (6.3%)	10 (12.7%)	

Diabetes mellitus; N (%)	Yes	68 (67.3%)	33 (32.7%)	0.45	17 (16.8%)	16 (15.8%)	0.22
	No	112 (71.8%)	44 (28.2%)		15 (9.6.%)	29 (18.6%)	

Past MI history; N (%)	Yes	49 (51.6%)	46 (48.4%)	<0.01	18 (18.9%)	28 (29.5%)	<0.01
	No	131 (80.9%)	31 (19.1%)		14 (8.6%)	17 (10.5%)	

Past CVA history; N (%)	Yes	37 (66.1%)	19 (33.9%)	0.46	8 (14.3%)	11 (19.6%)	0.76
	No	143 (71.1%)	58 (28.9%)		24 (11.9%)	34 (16.9%)	

Arrhythmia; N (%)	Yes	16 (51.6%)	15 (48.4%)	0.02	4 (12.9%)	11 (35.5%)	0.02
	No	164 (72.6%)	62 (27.4%)		28 (12.4%)	34 (15.0%)	

Anticoagulant consumption; N (%)	Yes	8 (72.7%)	3 (27.3%)	0.84	1 (9.1%)	2 (18.2%)	0.95
	No	172 (69.9%)	74 (30.1%)		31 (12.6%)	43 (17.5%)	

Hospitalization duration, days; mean (SD)		5.84 (4.28)	6.45 (4.14)	0.29^*∗∗∗*^	6.93 (4.20)	6.11 (4.12)	0.39^*∗∗∗∗*^

Hospitalization duration	<1 week	133 (72.7%)	50 (27.3%)	0.15	17 (9.3%)	33 (18.0%)	0.054
	≥1 week	47 (63.5%)	27 (36.5%)		15 (20.3%)	12 (16.2%)	

Outcome	Alive	168 (70.9%)	69 (29.1%)	0.31	27 (11.4%)	42 (17.7%)	0.21
	Expired	12 (60.0%)	8 (40.0%)		5 (25.0%)	3 (15.0%)	

^*∗*^by the Mann–Whitney *U* test, ^*∗∗*^ by the Kruskal–Wallis test, ^*∗∗∗*^ by the independent-sample *t*-test, ^*∗∗∗∗*^ by the one-way ANOVA test. EF: ejection fraction; CI: confidence interval; N: number; SD: standard deviation; MI: myocardial infarction; CVA: cerebrovascular accident.

**Table 2:** Association of demographic and risk factor variables with heart failure in logistic regression analysis among ischemic stroke patients.

Variables	Crude model		Adjusted model ^*∗*^	
	OR (95% CI)	*P*-value	OR (95% CI)	*P*-value
Age (years)	1.03 (1.01–1.05)	0.011	1.02 (1.00–1.05)	0.080
Male sex	1.25 (0.71–2.18)	0.437	—	—
Hyperlipidemia	1.81 (0.92–3.54)	0.084	1.61 (0.77–3.36)	0.209
Hypertension	2.28 (1.20–4.33)	0.012	1.74 (0.88–3.44)	0.110
Diabetes mellitus	1.24 (0.72–2.13)	0.445	—	—
Past MI history	3.97 (2.26–6.96)	<0.001	3.25 (1.82–5.81)	<0.001
Past CVA history	1.27 (0.67–2.38)	0.464	—	—
Arrhythmia	2.48 (1.16–5.32)	0.020	1.84 (0.80–4.26)	0.155
Anticoagulant consumption	0.87 (0.23–3.38)	0.842	—	—
Hospitalization duration (days)	1.03 (0.97–1.10)	0.288	—	—

^*∗*^ *P*-values less than 0.2 in the univariate model were included in the multivariate (adjusted) model.

## Data Availability

The datasets generated and analyzed during the current study are not publicly available; however, the data can be shared for research and authentication purposes upon reasonable request.
